# Detection of factors related to treatment reduction in docetaxel and ramucirumab for non-small cell lung cancer treatment

**DOI:** 10.1038/s41598-023-46775-9

**Published:** 2023-11-09

**Authors:** Yoshitaka Saito, Shinya Tamaki, Daisuke Hirate, Shinya Takada, Kenta Takahashi, Yoh Takekuma, Jun Sakakibara-Konishi, Yasushi Shimizu, Ichiro Kinoshita, Mitsuru Sugawara

**Affiliations:** 1 Department of Pharmacy, Hokkaido University Hospital, Kita 14-jo, Nishi 5-chome, Kita-ku, Sapporo, 060-8648 Japan; 2 Department of Clinical Pharmaceutics & Therapeutics, Faculty of Pharmaceutical Sciences, Hokkaido University of Science, 4-1, Maeda 7-jo 15-chome, Teine-ku, Sapporo, 006-8585 Japan; 3 Department of Pharmacy, KKR Sapporo Medical Center, 3-40, Hiragishi 1-jo 6-chome, Toyohira-ku, Sapporo, 062-0931 Japan; 4 Department of Pharmacy, Teine Keijinkai Hospital, 1-40, Maeda 1-jo 12-chome, Teine-ku, Sapporo, 006-8555 Japan; 5 Department of Pharmacy, National Hospital Organization Hokkaido Cancer Center, 3-4 Kikusui, Shiroishi-ku, Sapporo, 003-0804 Japan; 6 Department of Pharmacy, NTT Medical Center Sapporo, Minami 1-jo, Nishi 15-chome, Tyuou-ku, Sapporo, 060-0061 Japan; 7 Department of Respiratory Medicine, Faculty of Medicine, Hokkaido University, Kita 15-jo, Nishi 7-chome, Kita-ku, Sapporo, 060-8638 Japan; 8 Department of Medical Oncology, Faculty of Medicine and Graduate School of Medicine, Hokkaido University, Kita 15-jo, Nishi 7-chome, Kita-ku, Sapporo, 060-8638 Japan; 9 Laboratory of Pharmacokinetics, Faculty of Pharmaceutical Sciences, Hokkaido University, Kita 12-jo, Nishi 6-chome, Kita-ku, Sapporo, 060-0812 Japan

**Keywords:** Chemotherapy, Cancer therapy, Lung cancer

## Abstract

Treatment using docetaxel (DOC) and ramucirumab (RAM) is an effective regimen in second or later line advanced non-small cell lung carcinoma (NSCLC) treatment. However, it induces severe adverse effects, resulting in treatment reduction such as dose reduction and/or discontinuation. This study aimed to reveal the factor(s) associated with treatment reduction in DOC + RAM. We retrospectively evaluated patients with advanced NSCLC (n = 155). Treatment reduction of the second course due to severe adverse effects was conducted in 25.8% of the participants, and relative dose intensity at the second course was 95.7 ± 8.4% for DOC and 91.9 ± 24.8% for RAM. Multivariate logistic regression analyses identified that baseline anemia and prophylactic granulocyte colony-stimulating factor (G-CSF) administration are preventive factors for the reduction (adjusted odds ratio, 0.29; 95% confidence interval, 0.12–0.66; *P* = 0.004 for baseline anemia, 0.18; 0.08–0.42; *P* < 0.0001 for prophylactic G-CSF administration). The primary cause of the reduction was febrile neutropenia, and the same factors were identified. Our study revealed that patients with baseline anemia and prophylactic G-CSF administration have less risk for treatment reduction in DOC + RAM for NSCLC treatment.

## Introduction

Lung cancer is a leading cause of cancer-related death worldwide, with an increasing number of patients^1,2^. Non-small cell lung carcinoma (NSCLC) accounts for approximately 85% of all lung cancer^2^, and chemotherapy is the main strategy for advanced NSCLC treatment^3^.

Docetaxel (DOC) and ramucirumab (RAM) therapy is one of the effective regimens in second or later line NSCLC treatment^4,5^. DOC monotherapy used to be the representative second-line regimen for NSCLC treatment, and its dose limiting toxicity (DLT) is known to be neutropenia^6^. RAM binds to the extracellular domain of the vascular endothelial growth factor (VEGF) receptor-2 with high affinity, leading to inhibition of responses resulting from the activation of VEGFR-2 by VEGF-A, C and D^7^. In contrast, a combination of these medicines induces stronger adverse effects, such as hematotoxicities, febrile neutropenia (FN), anorexia, oral mucositis, and peripheral edema, compared to DOC monotherapy, and its DLT is reportedly hematotoxicity^4,5^. Moreover, as this treatment is normally conducted in outpatient settings, these adverse effects can be difficult to manage. Therefore, this treatment is recommended for patients younger than 75 years old and with a performance status (PS) of 0–1 in Japan, considering adverse effects^3^. Severe symptoms significantly reduce patients’ quality of life (QOL) and can cause dose reduction and/or discontinuation of these medicines. Risk factors of DOC-induced neutropenia are reportedly advanced aging, leucopenia, neutropenia, serum creatinine elevation, and serum albumin decrease at baseline, and a history of localized radiation therapy^8–10^. In contrast, the risk factors for the development of severe adverse effects causing treatment reduction such as dose reduction and/or discontinuation in this regimen are still unclear.

This study aimed to reveal the risk factor(s) for treatment reduction due to severe adverse effects in DOC + RAM therapy to improve treatment management.

## Results

### Patient characteristics

**Figure 1:**
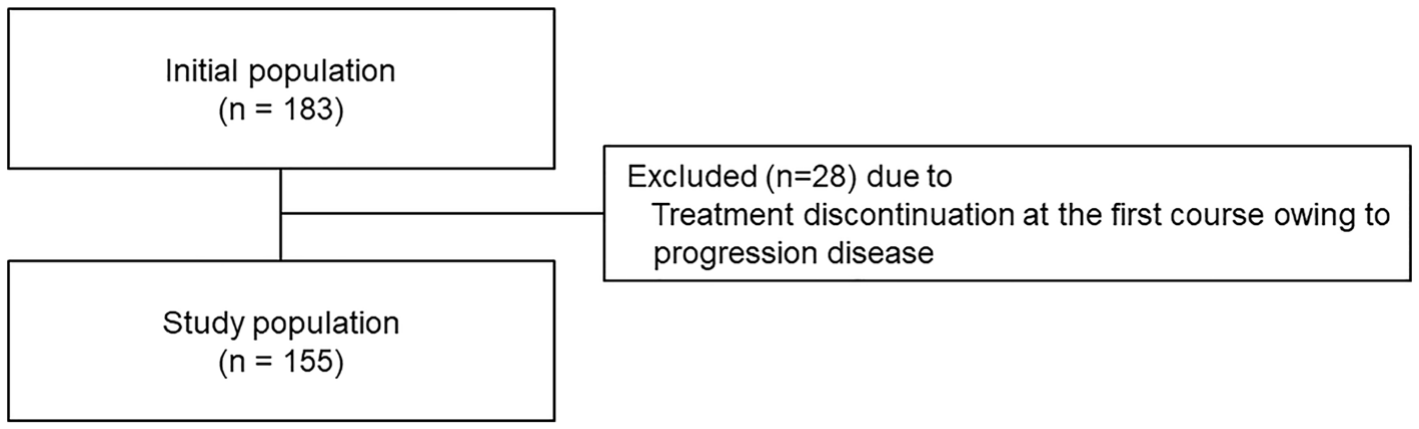
Flow diagram of the cohort and exclusions.

In total, 155 out of 183 patients were enrolled in this study, according to the eligibility criteria (Fig. 1). The baseline patient characteristics are shown in Table 1. Approximately two-thirds of the patients were male, and the median age was 65 years (range 33–80 years). The proportion of patients with lower neutrophil, hemoglobin, and platelet levels at baseline accounted for 5.8%, 64.5%, and 6.5%, respectively. The median serum albumin level was 3.9 g/dL (2.1–4.7 g/dL), and patients with hypoalbuminemia accounted for 67.7%. Approximately 80% were former or current smokers. Almost all patients had received a cytotoxic chemotherapeutic regimen prior to DOC + RAM therapy. Ten out of 155 patients (6.5%) received dose-reduced treatment from the treatment initiation. Prophylactic granulocyte colony-stimulating factor (G-CSF) administration from the first course was performed in 68.4% of the patients, and all received pegfilgrastim as prophylactic G-CSF in this study.

**Table 1:** Patient characteristics.

Sex (male/female)	105/50
Age (median, range)	65 (33–80)
ECOG performance status (n, %)	
0–1	135 (87.1%)
2	8 (5.2%)
Unknown	12 (7.7%)
Staging	
IV	124 (80.0%)
Recurrence	31 (20.0%)
Histology (n, %)	
Adenocarcinoma	131 (84.5%)
Squamous	19 (12.3%)
Others	4 (2.6%)
Unknown	1 (0.7%)
Liver metastasis existence (n, %)	22 (14.2%)
BSA (m^2^) (median, range)	1.67 (1.28–2.20)
BMI (kg/m^2^) (median, range)	22.53 (15.51–36.19)
Neutrophil (/μL) (median, range)	3,640 (1,020–14,008)
Number of less than LLN (n, %)	9 (5.8%)
Hb (g/dL) (median, range)	12.0 (7.3–17.6)
Number of less than LLN (n, %)	100 (64.5%)
Plt (× 10^3^/μL) (median, range)	249 (128–627)
Number of less than LLN (n, %)	10 (6.5%)
Albumin (g/dL) (median, range)	3.9 (2.1–4.7)
Number of less than LLN (n, %)	105 (67.7%)
Liver dysfunction (n, %)	36 (23.2%)
CCr (mL/min) (median, range)	71.9 (40.1–156.9)
Number of CCr less than 60 mL/min (n, %)	37 (23.9%)
Smoking history (n, %)	
Never	32 (20.6%)
Current smoker	14 (9.0%)
Former smoker	109 (70.3%)
Alcohol intake (≥ 5 days in a week) (n, %)	67 (43.2%)
Treatment line (n, %)	
2nd line	61 (39.4%)
3rd line	64 (41.3%)
4th line	19 (12.3%)
5th or later line	11 (7.1%)
Number of prior cytotoxic regimens (n, %)	
0	2 (1.3%)
1	118 (76.1%)
2	25 (16.1%)
3 or more	10 (6.5%)
Prior ICI administration (n, %)	79 (51.0%)
Dose reduction from initiation (n, %)	10 (6.5%)
Prophylactic G-CSF administration (n, %)	106 (68.4%)

ECOG, Eastern Cooperative Oncology Group; BSA, body surface area; BMI, body mass index; LLN, lower limit of normal; Hb, hemoglobin; Plt, platelet; CCr, creatinine clearance; ICI, immune checkpoint inhibitor; G-CSF, granulocyte colony-stimulating factor.

Liver dysfunction: grade 1 or higher aspartate aminotransferase, alanine aminotransferase, or total bilirubin elevation.

### Frequency of treatment reduction of the second course and adverse effect incidence at the first course

Treatment reduction of the second course due to severe adverse effects at the first course, which was the primary target of the present study, was conducted in 25.8% of the participants (Table 2). This included DOC dose reduction to 80% dosage alone for 50.0% of patients, dose reduction of both medicines (80% dose) for 25.0% of patients, and RAM discontinuation for 20.0% of patients. The adverse effects causing the reduction were FN (50.0%), grade 4 neutropenia (15.0%), bleeding (7.5%), oral mucositis (5.0%), and fluid retention (5.0%). Relative dose intensity (RDI) at the second course was 95.7 ± 8.4% for DOC and 91.9 ± 24.8% for RAM.

**Table 2:** Frequency of treatment reduction of the second course and its reduction causes, severe neutropenic symptoms at the first course of DOC + RAM treatment, and RDI of DOC and RAM.

Treatment reduction of the second course (n, %)	40 (25.8%)
Dose reduction of DOC to 80% dosage alone	20 (50.0%)
Dose reduction of both medicines to 80% dosage	10 (25.0%)
Discontinuation of RAM alone	8 (20.0%)
Dose reduction of RAM alone to 80% dosage	1 (2.5%)
Dose reduction of DOC (80%) with RAM discontinuation	1 (2.5%)
Treatment reduction due to	
Febrile neutropenia	20 (50.0%)
Neutropenia	6 (15.0%)
Bleeding	3 (7.5%)
Oral mucositis	2 (5.0%)
Fluid retention	2 (5.0%)
Multiple adverse effects beyond tolerability	2 (5.0%)
Thrombopenia	1 (2.5%)
Hand foot skin reaction	1 (2.5%)
Fatigue	1 (2.5%)
Decrease of performance status	1 (2.5%)
Hepatotoxicity	1 (2.5%)
Frequency of febrile neutropenia at the first course (n, %)	26 (16.8%)
Frequency of grade 3/4 neutropenia at the first course (n, %)	61 (39.4%)
Prophylactic G-CSF administration from the second course	33 (21.3%)
RDI at the first course (%, mean ± SD)	
DOC	98.8 ± 5.2
RAM	99.3 ± 3.5
RDI at the second course (%, mean ± SD)	
DOC	95.7 ± 8.4
RAM	91.9 ± 24.8

DOC, docetaxel; RAM, ramucirumab; G-CSF, granulocyte colony-stimulating factor; RDI, relative dose intensity; SD, standard deviation.

### Univariate and multivariate analyses of factors related to treatment reduction of the second course and FN incidence at the first course

Table 3A shows the results of the univariate and multivariate logistic regression analyses performed to identify factors associated with the treatment reduction at the second course. Baseline anemia and prophylactic G-CSF administration were detected as preventive factors for the reduction (adjusted odds ratio, 0.29; 95% confidence interval, 0.12–0.66; *P* = 0.004 for baseline anemia, 0.18; 0.08–0.42; *P* < 0.0001 for prophylactic G-CSF administration, respectively, Table 3A). As described previously, the primary cause of the reduction was FN, and Table 3B shows the results of the analyses regarding its incidence, resulting in the same factors being identified (0.22; 0.07–0.65; *P* = 0.006 for baseline anemia, 0.05; 0.01–0.16; *P* < 0.0001 for prophylactic G-CSF use).

**Table 3:** Univariate and multivariate logistic regression analyses of the risk factors associated with (A) treatment reduction from the second course and (B) febrile neutropenia incidence at the first course of DOC + RAM treatment.

(A)	Univariate analysis		Multivariate analysis	
	Odds ratio (95% CI)	*P*-value	Odds ratio (95% CI)	*P*-value
Sex				
Male/female	0.85 (0.40–1.81)	0.67	Excluded	–
Age (years)				
≥ 65/ < 65	1.43 (0.69–2.95)	0.34	Excluded	–
ECOG performance status				
0–1/2	2.45 (0.29–20.62)	0.41	Excluded	–
Clinical stage				
IV/recurrence	0.67 (0.28–1.58)	0.36	Excluded	–
Histology				
Adenocarcinoma/others	0.77 (0.29–2.03)	0.60	Excluded	–
Liver metastasis existence				
Present/absent	0.82 (0.28–2.40)	0.72	Excluded	–
BSA (m^2^)				
≥ 1.6/< 1.6	1.03 (0.49–2.17)	0.93	Excluded	–
Neutropenia				
Present/absent	0.34 (0.04–2.83)	0.32	Excluded	–
Anemia				
Present/absent	0.25 (0.12–0.52)	0.0003**	0.29 (0.12–0.66)	0.004**
Thrombopenia				
Present/absent	1.25 (0.31–5.09)	0.75	Excluded	–
Liver dysfunction				
Present/absent	1.37 (0.60–3.11)	0.46	Excluded	–
Renal dysfunction				
Present/absent	1.09 (0.47–2.51)	0.85	Excluded	–
Hypoalbuminemia				
Present/absent	0.54 (0.26–1.15)	0.11	0.89 (0.37–2.11)	0.79
Smoking history				
Current or former/never	0.49 (0.21–1.13)	0.09	0.46 (0.18–1.20)	0.11
Alcohol intake (≥ 5 days in a week)				
Present/absent	0.63 (0.30–1.33)	0.22	Excluded	–
Treatment line				
Second-line/third- or later-line	1.04 (0.50–2.17)	0.92	Excluded	–
Number of prior cytotoxic regimens				
0–1/2 or more	1.23 (0.51–2.98)	0.65	Excluded	–
ICI treatment history				
Present/absent	0.63 (0.31–1.31)	0.22	Excluded	–
Dose reduction from initiation				
Present/absent	0.70 (0.14–3.46)	0.67	Excluded	–
Prophylactic G-CSF administration				
Present/absent	0.19 (0.09–0.40)	< 0.0001**	0.18 (0.08–0.42)	< 0.0001**

(B)	Univariate analysis		Multivariate analysis	
	Odds ratio (95% CI)	*P*-value	Odds ratio (95% CI)	*P*-value
Sex				
Male/female	1.36 (0.53–3.48)	0.52	Excluded	–
Age (years)				
≥ 65/< 65	0.79 (0.34–1.85)	0.59	Excluded	–
ECOG performance status				
0–1/2	1.29 (0.15–11.03)	0.82	Excluded	–
Clinical stage				
IV/recurrence	1.06 (0.37–3.08)	0.91	Excluded	–
Histology				
Adenocarcinoma/others	0.96 (0.30–3.09)	0.94	Excluded	–
Liver metastasis existence				
Present/absent	1.12 (0.35–3.63)	0.85	Excluded	–
BSA (m^2^)				
≥ 1.6/< 1.6	1.83 (0.72–4.67)	0.20	Excluded	–
Neutropenia				
Present/absent	0.61 (0.07–5.06)	0.64	Excluded	–
Anemia				
Present/absent	0.22 (0.09–0.54)	0.0009**	0.22 (0.07–0.65)	0.006**
Thrombopenia				
Present/absent	1.26 (0.25–6.31)	0.78	Excluded	–
Liver dysfunction				
Present/absent	1.60 (0.63–4.07)	0.32	Excluded	–
Renal dysfunction				
Present/absent	1.53 (0.60–3.88)	0.37	Excluded	–
Hypoalbuminemia				
Present/absent	0.49 (0.21–1.15)	0.10	0.88 (0.30–2.60)	0.81
Smoking history				
Current or former/never	1.15 (0.49–2.69)	0.74	Excluded	–
Alcohol intake (≥ 5 days in a week)				
Present/absent	0.63 (0.30–1.33)	0.22	Excluded	–
Treatment line				
Second-line/third- or later-line	1.16 (0.49–2.72)	0.74	Excluded	–
Number of prior cytotoxic regimens				
0–1/2 or more	0.75 (0.29–1.97)	0.56	Excluded	–
ICI treatment history				
Present/absent	0.66 (0.28–1.54)	0.34	Excluded	–
Dose reduction from initiation				
Present/absent	Enable to calculate	–	Excluded	–
Prophylactic G-CSF administration				
Present/absent	0.05 (0.02–0.15)	< 0.0001**	0.05 (0.01–0.16)	< 0.0001**

Liver dysfunction: grade 1 or higher aspartate aminotransferase, alanine aminotransferase, or total bilirubin elevation.

Renal dysfunction: creatinine clearance of < 60 mL/min.

CI, confidence interval; ECOG, Eastern Cooperative Oncology Group; BSA, body surface area; ICI, immune checkpoint inhibitor; G-CSF, granulocyte colony-stimulating factor.

**P < 0.01.

### Relative dose intensity at the second course in patients with baseline anemia and G-CSF prophylaxis

We also assessed the influence of baseline anemia and prophylactic G-CSF administration on the RDI of DOC and RAM at the second course (Table 4). Treatment reduction was conducted in 16.0% and 43.6% of the patients with and without baseline anemia (*P* = 0.0003), and 15.1% and 49.0% of those with and without prophylactic G-CSF administration (*P* < 0.0001), respectively. The median RDI of DOC at the second course in patients with and without baseline anemia was 97.8% and 91.8%, and that in prophylactic G-CSF administration was 97.7% and 91.2%, respectively, with significant differences (*P* < 0.0001 for both). In contrast, median RAM RDI was 93.3% and 89.5% in baseline anemia, which was not significant (*P* = 0.18), although a significant difference was identified in prophylactic G-CSF use (95.8% vs. 83.4%, respectively, *P* = 0.002).

**Table 4:** Relative dose intensity of DOC and RAM in patients with or without baseline anemia and prophylactic G-CSF administration.

	Treatment reduction (n, %)	*P*-value	DOC RDI (%)	*P*-value	RAM RDI (%)	*P*-value
Baseline anemia						
Present (n = 100)	16 (16.0%)	< 0.01**	97.8 ± 6.3	< 0.01**	93.3 ± 24.0	0.18
Absent (n = 55)	24 (43.6%)		91.8 ± 10.2		89.5 ± 26.3	
Prophylactic G-CSF administration						
Present (n = 106)	16 (15.1%)	< 0.01**	97.7 ± 6.4	< 0.01**	95.8 ± 19.3	< 0.01**
Absent (n = 49)	24 (49.0%)		91.2 ± 10.3		83.4 ± 32.5	

DOC, docetaxel; RDI, relative dose intensity; RAM, ramucirumab; G-CSF, granulocyte colony-stimulating factor.

**P < 0.01.

### Impact of baseline anemia on the treatment reduction from the second course and FN incidence at the first course in patients who were not administered prophylactic G-CSF

Table 5 presents the influence of baseline anemia on the treatment reduction from the second course and FN incidence at the first course in patients without prophylactic G-CSF administration. Patients with baseline anemia experienced treatment reduction and FN at a significantly lower rate than those without anemia (29.6% and 72.7% in patients with and without anemia, *P* = 0.004 for dose reduction, and 29.6% and 63.6%, *P* = 0.02, for FN incidence).

**Table 5:** Impact of baseline anemia on the treatment reduction from the second course and FN incidence at the first course, in patients who were not administered prophylactic G-CSF.

	Treatment reduction from the second course (n, %)	*P*-value	Incidence of FN at the first course (n, %)	*P*-value
Baseline anemia				
Present (n = 27)	8 (29.6%)	0.004**	8 (29.6%)	0.02*
Absent (n = 22)	16 (72.7%)		14 (63.6%)	

FN, febrile neutropenia.

*P < 0.05, **P < 0.01.

## Discussion

DOC + RAM therapy is one of the most effective regimens in the second or later line NSCLC treatment. However, its administration induces severe adverse effects. In the REVEL study, adverse effects such as FN, neutropenia, thrombocytopenia, anorexia, oral mucositis, and peripheral edema occurred more strongly in treatments with DOC + RAM compared to DOC + placebo, leading to higher dose delays and reduction (42% vs. 32%, and 29% vs. 21%, respectively)^4,11^. In addition, some adverse effects such as hematotoxicities, FN, anorexia, oral mucositis, and peripheral edema appeared in higher rates and increased in severity in Japanese patients compared to non-Japanese patients registered in the REVEL study including approximately 10% Asians, although DOC dosage in Japanese patients was lower (60 mg/m^2^)^4,5^. Similar results were confirmed in gemcitabine + nanoparticle albumin-bound paclitaxel treatment for pancreatic cancer^12^. Kenmotsu et al. suggested that Japanese patients seemed to be more susceptible to DOC-induced toxicities^6^. They speculated that this could be caused by unknown genetic factors, higher sensitivity to adverse effects, differences in unbound docetaxel concentrations, or baseline counts of white blood cells^6^. In addition, Choi et al. suggested that *ABCB1* (2677G/T) and *SLCO1B3* (rs11055585) might be major genetic predictors of DOC-related toxicities in DOC-containing chemotherapy^13^. We consider that multiple factors, such as differences in single nucleotide polymorphism in transporters and cytochrome P450 (CYP) and higher sensitivity to the adverse effects in the Japanese population, might have caused the differences. Therefore, the results of the present study should be interpreted considering this possibility.

Treatment reduction was performed in 25.8% of the patients who received at least two courses of the treatment, and the most problematic adverse effect inducing the reduction was FN. Logistic regression analyses reveled that baseline anemia and prophylactic G-CSF use were preventive factors for the treatment reduction and FN development.

Several reports have also shown that prophylactic pegfilgrastim administration significantly reduces FN incidence to 0–5% in this regimen^14–16^. On the other hand, pegfilgrastim administration reportedly worsens taxane-associated acute pain syndrome (T-APS), although it can be controlled by analgesics such as non-steroidal anti-inflammatory drugs or acetaminophen^17^. However, T-APS is taxane-dose-dependent symptoms^17,18^, and its incidence in 60 mg/m^2^ DOC is not that problematic. Consequently, prophylactic G-CSF administration with careful attention to T-APS could be one of the best strategies for safer and more efficient DOC + RAM provision.

Interestingly, baseline anemia was detected as a preventive factor, although previous reports suggested it as a risk factor for severe neutropenia development in other chemotherapeutic treatments^19–22^. Additionally, we compared patient backgrounds between those with and without baseline anemia and found that males and those with hypoalbuminemia were more likely to be included as patients with anemia (Supplemental Table 1). However, sex and hypoalbuminemia were not associated with severe adverse effects in the logistic analysis. Additionally, patients without baseline anemia significantly experienced more severe symptoms compared to those with anemia in the non-prophylactic G-CSF population. These results suggest that baseline anemia can induce less severe adverse effects in DOC + RAM for NSCLC. There is no report evaluating factors associated with the overall severe adverse effects inducing treatment reduction in DOC + RAM; however, there are some studies regarding FN in DOC monotherapy. Uchida et al. have reported that higher baseline hemoglobin levels could be associated with FN development in patients with NSCLC, although it was not assessed in multivariate analysis owing to the small patient population (unadjusted odds ratio 1.34; 95% confidence interval, 0.99–1.88; *P* = 0.06)^23^. In contrast, Hirasawa et al. and Kwon et al. reported that baseline hemoglobin levels are not associated in patients with castration-resistant prostate cancer^8,9^. However, the patient populations for NSCLC and prostate cancer are notably distinct, which can explain the disparity between the observations. Consequently, further evaluation is required to confirm the results.

Several studies have suggested that anemia and the effect of VEGF or VEGF inhibitors are related, but this remains controversial^24–30^. Dunst et al. reported that tissue hypoxia is a major stimulus for the upregulation of VEGF via impairment of tissue oxygenation, suggesting that anemia may promote tumor angiogenesis via hypoxia^26,27^. Krzystek-Korpacka et al. also reported that mild pre-treatment anemia is associated with cancers metastasizing, especially to regional lymph nodes, which seems to be mediated by angiogenic factors^29^. Furthermore, Lim et al. reported that vascular dilation through VEGFR2 signaling is the mechanism underlying VEGF-induced bone marrow mobilization and anemia^30^. In addition, vascular normalization by VEGF blockade induces a pressure gradient across the vasculature and improves drug penetration in tumors^31,32^. Consequently, it is possible that anemia progressed tumor angiogenesis, leading to the elevation of DOC delivery to tumors by RAM efficacy, reducing the delivery to other organs, which resulted in a decrease in severe adverse effects. However, further studies are needed to elucidate the mechanisms.

As previously mentioned, some baseline factors are reportedly associated with DOC-induced neutropenia and FN^8–10,23^. However, all previously reported factors were not identified as factors in DOC + RAM treatment; RAM addition significantly induces more severe adverse effects, such as FN and/or neutropenia^4,5^, which has significantly affected the non-identification of these factors.

Consequently, prophylactic pegfilgrastim should be administered and adverse effects should be cautiously monitored, particularly in the first course of treatment in patients with normal baseline hemoglobin levels.

The present study had some limitations. First, this study was a retrospective evaluation with a relatively small patient population. Particularly, we consider that evaluation of factors for FN incidence was insufficient owing to small number of the events. Second, we assessed risk factors during the first course, as the treatment dosage and supportive care would generally be structured by referring to the degree of adverse effects in the first course. In contrast, multiple courses of the treatment can result in more severe adverse effects, such as peripheral edema, neuropathy, and proteinuria. Therefore, evaluation of all treatment courses is needed, and the influence of dose reduction due to cumulative toxicities must be considered. Third, we did not evaluate the treatment efficacy. Finally, we did not assess polymorphisms of drug-metabolizing enzymes or transporters such as CYP3A4, CYP3A5, ABCB1, ABCC2, and SLCO1B3, which can impact the pharmacokinetics of DOC^6^. Moreover, there may be unknown factors that enhance the RAM-intensifying effect of DOC-induced adverse effects. Thus, our preliminary findings should be validated in future research.

In conclusion, our study revealed that patients with baseline anemia and prophylactic G-CSF administration have less risk for treatment reduction in DOC + RAM for NSCLC treatment. This treatment highly induces severe adverse effects, leading to treatment reduction. Therefore, further evaluation for the prophylaxis is needed.

## Methods

### Patients

In this retrospective multicenter observational study, we evaluated 183 patients with advanced NSCLC who received the DOC + RAM regimen from September 2016 to October 2021. All patients met the following baseline criteria: (1) age ≥ 20 years old; (2) detailed patient information available from medical records; (3) 0–2 Eastern Cooperative Oncology Group PS (ECOG-PS); (4) absolute neutrophil count ≥ 1.0 × 10^3^ cells/µL and platelet count ≥ 1.0 × 10^5^ cells/µL; and (5) sufficient renal or liver function for treatment induction. Patients who discontinued the treatment in the first course due to disease progression or transferred hospitals during chemotherapy were excluded.

We calculated the number of final patients to be approximately 150, based on the assumption that approximately 30% of the patients received dose reduction at the second course by reference to the previous reports and our clinical experience^4,5^. We tried to include approximately four covariates in the multivariate logistic regression analysis. The present study was approved by the institutional review board of each participating institution (in case of Hokkaido University Hospital, approval number was 020–0366) and was conducted in accordance with the Declaration of Helsinki and STROBE statement.

### Treatment methods

DOC (60 mg/m^2^) and RAM (10 mg/kg) were intravenously administered every 3 weeks^4,5^. Dexamethasone 6.6 mg was administered before the chemotherapeutic agents for premedication. Treatment was reduced or discontinued by the physician’s decision according to the criteria in the previous reports^4,5^.

### Evaluation of adverse effects and treatment reduction

The required information was obtained from the patients’ medical records. The primary endpoint of this study was defined as detection of the risk factor(s) for the frequency of treatment reduction, such as dose reduction and/or discontinuation at the second course owing to severe adverse effects at the first course. The secondary endpoints were the elucidation of the risk factor(s) associated with the development of FN during the first course of treatment and comparison of the rate of treatment reduction and FN incidence, and RDI between the specific populations. Adverse effects were evaluated in accordance with the Common Terminology Criteria for Adverse Events version 5.0.

### Statistical analysis

Univariate and multivariate logistic regression analyses were conducted to determine the independent factors associated with the frequency of treatment reduction and FN, using the following baseline covariates: sex, age, ECOG-PS, clinical staging, histology, liver metastasis existence, body surface area (BSA), neutropenia, anemia, thrombopenia, liver dysfunction (grade 1 or higher aspartate aminotransferase, alanine aminotransferase, or total bilirubin elevation), renal dysfunction (creatinine clearance calculated by the Cockcroft-Gault formula of < 60 mL/min), hypoalbuminemia, smoking history, regular alcohol intake (≥ 5 days in a week), treatment line, number of prior cytotoxic regimens, immune checkpoint inhibitor treatment history, dose reduction from treatment initiation, and prophylactic G-CSF administration. Variables that had potential associations, as suggested by univariate logistic regression analysis (*P* < 0.20), were considered when building the multivariable model. The RDI and treatment reduction frequency between the specific patient groups were compared using Student’s t-test and Fisher’s exact probability test, respectively. All analyses were performed using JMP version 16.1 statistical software (SAS Institute Inc.). Differences were considered statistically significant when *P*-values were < 0.05.

### Ethics approval and consent to participate

All procedures performed in this study were carried out in accordance with the ethical standards of the institutional and/or national research committee and the 1964 Helsinki Declaration and its later amendments or comparable ethical standards. The study was approved by the institutional review board of each participating institution (in case of Hokkaido University Hospital approval number: 020–0366). The requirement for formal consent for this type of study was waived by the Ethical Review Board for Life Science and Medical Research of Hokkaido University Hospital, Institutional Review Board of KKR Sapporo Medical Center, Teine Keijinkai Hospital, Hokkaido Cancer Center, and NTT Medical Center Sapporo.

### Supplementary Information

Supplementary Table 1.

## Data Availability

The datasets used and/or analyzed in the current study are available from the corresponding author upon reasonable request.
