# Supplementary material for: Detection of factors related to treatment reduction in docetaxel and ramucirumab for non-small cell lung cancer treatment
Source: Sci Rep. 2023 Nov 9;13:19457. doi: 10.1038/s41598-023-46775-9 (PMC10636055; doi:10.1038/s41598-023-46775-9)
Supplement: Supplementary file 1 — Supplementary Table 1. [file 41598_2023_46775_MOESM1_ESM.docx]

**Supplemental Table 1. Comparison of patient backgrounds between patients with and without baseline anemia**

|  | Patients with anemia (n=100) | Patients without anemia (n=55) | P-value |
| --- | --- | --- | --- |
| Sex  Male/Female | 77/23 | 28/27 | 0.001** |
| Age (years)  ≥65/<65 | 54/46 | 25/30 | 0.32 |
| ECOG-PS  0–1/2 | 84/7 | 51/1 | 0.26 |
| Clinical stage  Ⅳ/Recurrence | 79/21 | 45/10 | 0.83 |
| Histology  Adenocarcinoma/Others | 83/16 | 48/7 | 0.64 |
| Liver metastasis existence  Present/Absent | 16/84 | 6/49 | 0.47 |
| BSA (m^2^)  ≥1.6/<1.6 | 63/37 | 33/22 | 0.73 |
| Neutropenia  Present/Absent | 7/93 | 2/53 | 0.49 |
| Anemia  Present/Absent | 100/0 | 0/55 | <0.0001** |
| Thrombopenia  Present/Absent | 7/93 | 3/52 | 1.00 |
| Liver dysfunction  Present/Absent | 22/78 | 14/41 | 0.69 |
| Renal dysfunction  Present/Absent | 26/74 | 11/44 | 0.44 |
| Hypoalbuminemia  Present/Absent | 77/23 | 28/27 | 0.001** |
| Smoking history  Current or former/Never | 83/17 | 40/15 | 0.15 |
| Alcohol intake Present/Absent | 45/55 | 22/33 | 0.61 |
| Treatment line  Second-line/Third- or later-line | 41/59 | 20/35 | 0.61 |
| Number of prior cytotoxic regimens  0–1/2 or more | 80/20 | 40/15 | 0.32 |
| ICIs treatment history  Present/Absent | 53/47 | 26/29 | 0.51 |
| Dose reduction from initiation  Present/Absent | 7/93 | 3/52 | 1.00 |
| Prophylactic G-CSF administration  Present/Absent | 73/27 | 33/22 | 0.11 |

***P*<0.01

ECOG-PS, Eastern Cooperative Oncology Group performance status; BSA, body surface area; ICIs, immune checkpoint inhibitors; G-CSF, granulocyte colony-stimulating factor.
